# Psychometric evaluation of the Oswestry Disability Index in patients with chronic low back pain: factor and Mokken analyses

**DOI:** 10.1186/s12955-017-0768-8

**Published:** 2017-10-03

**Authors:** Chin-Pang Lee, Tsai-Sheng Fu, Chia-Yih Liu, Ching-I Hung

**Affiliations:** 1 Department of Psychiatry, Chang Gung Memorial Hospital, Linkou, Taoyuan, Taiwan; 2 Department of Orthopaedics, Chang Gung Memorial Hospital, Linkou, Taoyuan, Taiwan; 3 School of Medicine, Chang Gung University, 5, Fusing Street, Gueishan District, Taoyuan, 33305 Taiwan

**Keywords:** The Oswestry Disability Index (ODI), Psychometrics, Validity, Reliability, Dimensionality, Low back pain

## Abstract

**Background:**

Disputes exist regarding the psychometric properties of the Oswestry Disability Index (ODI). The present study was to examine the reliability, validity, and dimensionality of a Chinese version of the ODI version 2.1 in a sample of 225 adult orthopedic outpatients with chronic low back pain [mean age (SD): 40.7 (11.4) years].

**Methods:**

We conducted reliability analysis, exploratory bifactor analysis, confirmatory factor analysis, and Mokken scale analysis of the ODI. To validate the ODI, we used the Short-Form 36 questionnaire (SF-36) and visual analog scale (VAS).

**Results:**

The reliability, and discriminant and construct validities of the ODI was good. The fit statistics of the unidimensional model of the ODI were inadequate. The ODI was a weak Mokken scale (H_s_ = 0.31).

**Conclusions:**

The ODI was a reliable and valid scale suitable for measurement of disability in patients with low back pain. But the ODI seemed to be multidimensional that was against the use of the raw score of the ODI as a measurement of disability.

## Background

Low back pain (LBP) is the second leading cause of disability in the world [1]. The level of disability in patients with LBP is an important outcome measure for clinical practice and research [2]. The Oswestry Disability Index (ODI) is one of the most commonly used scales that assess the disability related to LBP [2, 3].

The scoring of the ODI is the simple sum score of the items of the ODI multiplied by two [3]. The derived score of the ODI is a measurement of the level of disability related to LBP [3]. But the items of the ODI are ordinal measurements. To use the raw score of the ODI as a valid measurement, three assumptions are required: (1) the ODI is unidimensional; (2) all the items of the ODI are equally correlated with the measured construct (i.e. LBP-related disability); and (3) the point intervals are equal on the ODI [4]. But these assumptions are typically unchecked and unjustified [4]. Hence, to use the raw score of the ODI as a measurement, it is necessary to check whether the ODI meets these assumptions.

Disputes exist in the psychometric properties of the ODI. First, the construct and transcultural validities of the ODI are uncertain [5]. Second, the unidimensionality of the ODI is conflicting [6, 7]. Several items of the ODI poorly fit unidimensionality [6–8]. Third, the ODI appears to have a floor effect that the ODI poorly differentiates patients with little disability [6, 7, 9]. Fourth, the ODI also has a ceiling effect that limits differentiating patients with high disability [6, 7, 9]. Such disputes suggest the raw score of the ODI is problematic.

Lue et al. (2008) developed the Chinese version of the ODI 2.1, and claimed that the ODI was unidimensional solely based upon its Chronbach’s α was 0.90 [10]. Such argument is false because Cronbach’s α does not measure dimensionality. Cronbach’s α is a measure of the mean inter-item covariance and the number of items [11]. No clear relationship exists between Cronbach’s α and the dimensionality of a scale [11]. Lue et al. (2013) has shown that the ODI fitted the Rasch model, and they suggested that the ODI is a unidimensional scale [7]. But in that study [7], the majority (6 of 10) of the items of the ODI violated monotonicity, suggesting that the ODI might be a multidimensional scale.

The present study was to address two research questions of the Chinese version of the ODI 2.1:

1. Is the ODI a unidimensional scale?
2. Is the ODI reliable and valid for measuring disability in adult orthopedic outpatients with chronic LBP?

## Methods

### Participants

We re-analysed the data from a cross-sectional sample of adult orthopedic outpatients with chronic LBP in Taiwan [12]. This study was conducted in the general orthopedics clinic of the Chang Gung Memorial Hospital, Linkou, from August 2008 to November 2010, and was approved by the Institutional Review Board of the same hospital. Inclusion criteria were as follows: (1) 20–65 years of age, and (2) chronic LBP that was defined as LBP for at least 3 months. Exclusion criteria were as follows: (1) receiving antidepressant or antipsychotic medication during the preceding 4 weeks, (2) psychotic symptoms, (3) mental retardation, and (4) severe cognitive impairment. All participants gave written informed consent before study enrollment.

The sample consisted of 225 patients [mean age (SD): 40.7 (11.4) years; 103 (45.8%) females]. As for comorbid mental illness per the DSM-IV criteria [13], 49 (21.8%) had major depressive disorder and 52 (23.1%) had at least one anxiety disorder. 83 (36.9%) patients had severe LBP.

### Instruments

#### The Oswestry Disability Index (ODI)

The ODI consists of 10 items on the degree of severity to which back (or leg) trouble has affected the ability to manage in everyday life [3]. The 10 sections cover the pain and the daily function (including pain intensity, personal hygiene, lifting, walking, sitting, standing, sleeping, sexual activity, social activity, and traveling). Each item is rated on a 6-point scale (0–5); the higher score means the higher level of disability related to LBP. The present study used the traditional Chinese version of the ODI 2.1 [10].

#### The short-form 36 questionnaire (SF-36)

The SF-36 consists of 36 items for measuring the general health status of patients [14]. The SF-36 has 8 subscales as follows: physical functioning (PF), role limitations due to physical health problems (Role-physical, RP), bodily pain (BP), general health (GH), vitality (VT), social functioning (SF), role limitation due to emotional problems (Role-emotional, RE) and mental health (MH). Each subscale is rated on a scale of 0–100; the higher score means the better health status. We used the traditional Chinese version of the SF-36 [15]. We adopted two kinds of aggregate scoring of the SF-36. First, according to the Medical Outcomes Study conceptual model, the Physical Health score (PHS) is derived from the sum of PF, RP, BP, and GH; the Mental Health score (MHS) is derived from the sum of VT, SF, RE, and MH [14, 16]. Second, the standard physical component summary (PCS) and mental health component summary (MCS) scores were calculated with the formulae available in the study by Leese et al. [16] and the norms of Taiwan population [15]:

1. Standardized scores of the SF-36:

$$ PFZ=\frac{\left( PF-92.24\right)}{16.16}, RPZ=\frac{\left( RP-83.65\right)}{33.27}, BPZ=\frac{\left( BP-84.84\right)}{19.42}, GHZ=\frac{\left( GH-69.29\right)}{21.27}, VTZ=\frac{\left( VT-68.27\right)}{18.66}, SFZ=\frac{\left( SF-86.81\right)}{17.05}, REZ=\frac{\left( RE-79.4\right)}{36.07}, MHZ=\frac{\left( MH-73.01\right)}{16.55} $$

2. PCS_1_ and MCS_1_ scores:

$$ {PCS}_1=0.42402\times PFZ+0.35119\times RPZ+0.31754\times BPZ+0.24954\times GHZ+0.02877\times VTZ-0.00753\times SFZ-0.19206\times REZ-0.22069\times MHZ $$

$$ {MCS}_1=-0.22999\times PFZ-0.12329\times RPZ-0.09731\times BPZ-0.01571\times GHZ+0.23534\times VTZ+0.26876\times SFZ+0.43407\times REZ+0.4858\times MHZ $$

3. PCS and MCS scores:

$$ PCS={PCS}_1\times 10+50 $$

$$ MCS={MCS}_1\times 10+50 $$

### The visual analog scale (VAS)

Each patient rated the pain intensity of the back and lower legs on the VAS with a horizontal line of 10 cm [17]. In this study, VAS ≥ 7 was considered to indicate severe pain.

## Statistical analysis

We conducted all analyses in R version 3.3.1 [18]. The *P*-values were two-tailed with the significance level of 0.05. We summarized the item statistics of the ODI with the *likert* package [19].

### Reliability

To assess the reliability of the ODI, we used the *psych* package for getting four coefficients as follows: Cronbach’s α, Revelle’s β, and McDonald’s ω_t_ and ω_h_ [20, 21]. Revelle’s β refers to the worst split-half reliability. The coefficient ω_t_ refers to the amount of reliable variance in a scale. The coefficient ω_h_ refers to an estimate of the general factor saturation of a scale. As for each of the four coefficients of a scale, a value of 0.7 or higher marks good reliability. Also, the ordered sequence of the four coefficients offers insight into the dimensionality of a scale [21]. For a unidimensional scale, ω_h_ should be equal or greater than Chronbach’s α [21].

We took two steps to get the coefficients ω_t_ and ω_h_ of the ODI. First, we conducted parallel analysis of the ODI data to decide the proper number of extracted factors. Second, we conducted exploratory bifactor analysis (EBA) of the ODI data to get ω_t_, ω_h_, and the explained common variance (ECV) for the general factor. If the ECV is larger than 60%, a unidimensional construct is confirmed [22]. We obtained the corresponding 95% bias-corrected and accelerated bootstrap confidence intervals of α, β, ω_t_, ω_h_, and the ECV with 10,000 bootstrap replications with the *boot* package [23, 24].

### Convergent and Discriminant validities

To test the convergent validity of the unidimensional model of the ODI, we conducted confirmatory factor analysis (CFA) with the *lavaan* and *matrixpls* packages [25, 26]. If the composite reliability (CR) is greater than 0.7 and the average variance extracted (AVE) greater than 0.5, then the convergent validity is confirmed [27]. The cutoffs of model fit statistics are as follows: the root mean square error of approximation (RMSEA) < 0.06, the close fit (CFit) test that was non-significant (i.e., the probability value that the RMSEA ≤ 0.05 was greater than 0.05), the standardized root mean square residual (SRMR) ≤ 0.05, the weighted root mean square residual (WRMR) < 1.0, the comparative fit index (CFI) ≥ 0.95, and the Tucker-Lewis index (TLI) ≥ 0.95 [28–30].

To test the discriminant validity of the ODI, we used the Fornell-Larcker criterion and the heterotrait-monotrait (HTMT) ratio with the PCS and MCS of the SF-36 [16, 27, 31]. As for the Fornell-Larcker criterion, if the AVE of the ODI is larger than the squared correlations between the ODI and the PCS and MCS of the SF-36, then the discriminant validity of the ODI is confirmed. As for the HTMT ratio method, two criteria of discriminant validity are as follows: (1) the HTMT ratio should be less than 0.85 (HTMT_.85_), and (2) the 90% normal bootstrap confidence should not include 1 (HTMT_inference_) [23, 24, 31]. We got the corresponding 95% confidence intervals of CR, AVE, and HTMT ratios of the ODI with 10,000 bootstrap replications [23, 24].

### Mokken scale analysis (MSA)

MSA is one of non-parametric item response theory models and is useful for scrutinizing a scale [32, 33]. We conducted MSA of the ODI data with the *mokken* package [34, 35]. First, we got the three Loevinger’s scalability coefficients (*H*): item-pair (*H*_*ij*_), item (*H*_*i*_), and scale (*H*_*s*_) [34–36]. The rules of thumb for the *H* values are as follows: a scale is weak if 0.3 ≤ *H* < 0.4, moderate if 0.4 ≤ *H* < 0.5, and strong if *H* ≥ 0.5 [34–36]. Second, we examined local independence with conditional association procedure, monotonicity with item-rest regression, and non-intersection with the *restscore* method [32, 34, 35, 37, 38]. Finally, we assessed item ordering with the *manifest IIO* method and the backward selection procedure [34]. Next, we rated IIO of the selected items on the coefficient *H* ^*T*^ [39]. The rules of thumb for *H* ^*T*^ values are as follows: a weak IIO if 0.3 ≤ *H* ^*T*^ < 0.4, moderate if 0.4 ≤ *H* ^*T*^ < 0.5, and strong if *H* ^*T*^ ≥ 0.5. We rated the reliability of a Mokken scale on the latent class reliability coefficient (LCRC) [40]. A reliable scale should have a LCRC ≥ 0.7.

### Construct validity

We calculated the Pearson’s product-moment correlation coefficients between the ODI and the SF-36 aggregate scores [i.e. PHS, MHS, PCS, and MCS], and the VAS. As the ODI is a scale of LBP-related disability, we hypothesized that the absolute values of the correlation coefficients between the ODI and the physical domain (i.e. the PHS, PCS, and VAS) should be greater than those between the ODI and the mental domain (i.e. the MHS and MCS).

## Results

Table 1 shows the demographic and clinical characteristics of the sample. Among the 225 patients, 49 (21.8%) patients had major depressive disorder. Among them, 21 (42.9%) patients were in a current major depressive episode, 21 (42.9%) were in partial remission of depression, and 7 (14.3%) were in full remission. Among the 225 patients, 149 (66.2%) patients had abnormal radiographic findings. 96 (42.7%) patients had associated leg symptoms, including leg radiation pain, leg numbness, intermittent claudication, and neurological deficits. 68 (30.2%) patients had medical comorbidities. Table 2 shows the item statistics of the ODI. As for the combined proportion of response ≥3 (i.e. at least moderate disability), the items 1 (pain intensity), 6 (standing), and 9 (social activity) were the highest among the 10 items of the ODI. Table 3 shows the abridged summary of the ODI, SF-36, and VAS. Parallel analysis of the ODI data revealed that the number of factor extracted should be four. Table 4 shows the summary of the reliability and validity statistics of the ODI. The ODI had a Cronbach’s α, a McDonald’s ω_t_, and an LCRC greater than 0.7, indicating the ODI was reliable. The ODI was multidimensional according to the following criteria: (1) ω_h_ was lower Chronbach’s α; (2) the ECV was below 60%; and (3) the fit statistics of the unidimensional model of the ODI were inadequate. The ODI had poor discriminant validity with the PCS but good discriminant validity with the MCS of the SF-36. The ODI was a weak mokken scale (*H*_*s*_ = 0.31). Table 5 shows the correlation coefficients between the ODI and other scales. The ODI negatively correlated to the aggregate scores of the SF-36 and positively to the VAS. Table 6 shows the item scalability coefficients (*H*_*i*_) of the ODI. Each item of the ODI had no violation of local independence and monotonicity; also, no serious violation of non-intersection existed. The backward selection procedure of the ODI removed 3 items (# 3, 5, and 7). The other 7 items had weak IIO property (*H* ^*T*^ = 0.36). The hierarchical item ordering of the 7 items was the ascending order of the mean scores of these items [2 (personal hygiene), 10 (traveling), 4 (walking), 8 (sexual life), 9 (social life), and 1 (pain intensity)].

**Table 1:** Demographic and clinical characteristics of the sample (*N* = 225)

Characteristics	Statistics
Age, mean (SD), years	40.7 (11.4)
Female, N (%)	103 (45.8)
Education, mean (SD), years	11.4 (3.4)
Paid Employment, N (%)	152 (67.6)
Married, N (%)	156 (69.3)
Obesity (BMI ≥ 27 kg/m^2^), N (%)	47 (20.9)
Smoking, N (%)	70 (31.1)
Major depressive disorder, N (%)	49 (21.8)
At least one anxiety disorder, N (%)	52 (23.1)
Severe low back pain^a^, N (%)	83 (36.9)
Past operative history, N (%)	12 (5.3)
Abnormal radiographic findings, N (%)	149 (66.2)
Spondylosis, N (%)	141 (62.7)
Spondylolisthesis, N (%)	27 (12)
Scoliosis, N (%)	12 (5.3)
Other findings, N (%)	21 (9.3)
Associated leg symptoms^ab^, N (%)	96 (42.7)
Medical comorbidities, N (%)	68 (30.2)
Hyperlipidemia, N (%)	46 (20.4)
Hypertension, N (%)	27 (12.0)
Diabetes mellitus, N (%)	11 (4.9)

^a^Visual analog scale (VAS) ≥ 7

^b^Associated leg symptoms included leg radiation pain, leg numbness, intermittent claudication, and neurological deficits

Abbreviations: *BMI* Body Mass Index

**Table 2:** Item statistics of the ODI

Item	Description	Mean (SD)	0	1	2	3	4	5
1	Pain intensity	3.9 (1.5)	1%	28%	8%	25%	24%	15%
2	Personal hygiene	1.8 (0.9)	49%	22%	28%	1%	0%	0%
3	Lifting	2.6 (1.3)	11%	60%	7%	7%	13%	2%
4	Walking	2.3 (1.1)	28%	34%	19%	17%	1%	1%
5	Sitting	2.8 (1.3)	22%	16%	32%	21%	8%	1%
6	Standing	2.8 (1.4)	15%	40%	9%	21%	14%	1%
7	Sleeping	2.4 (1.3)	39%	15%	27%	15%	2%	3%
8	Sexual life	2.4 (1.6)	40%	28%	4%	10%	15%	2%
9	Social life	2.6 (1.4)	31%	27%	10%	24%	3%	4%
10	Traveling	2.2 (1.3)	28%	55%	5%	4%	2%	7%

**Table 3:** Abridged summary of the ODI, SF-36, and VAS

Scale	Mean	SD	Range
ODI	31.4	15.4	2–70
VAS			
Low back	5.0	2.3	0–10
Legs	3.7	2.4	0–10
SF-36			
PHS	193	74	32–359
MHS	234	95	9–400
PCS	31.9	9.9	4.1–55.0
MCS	45.9	13.6	8.1–70.6

Abbreviations: *ODI* the Oswestry Disability Index, *VAS* Visual Analog Scale, *SF-36* Short-Form 36 questionnaire, *PHS* Physical Health score, *MHS* Mental Health score, *PCS* Physical Component Summary, *MCS* Mental Health Component Summary

**Table 4:** Reliability and validity statistics of the ODI

Indicator	Statistics
Reliability	
Cronbach’s α [95% CI]	0.78 [0.74–0.82]
Revelle’s β [95% CI]	0.66 [0.56–0.73]
McDonald’s ω_h_ [95% CI]	0.58 [0.47–0.69]
McDonald’s ω_t_ [95% CI]	0.85 [0.80–0.87]
ECV [95% CI]	45% [36–58%]
CFA	
CR [95% CI]	0.84 [0.81–0.86]
AVE [95% CI]	0.35 [0.31–0.39]
Fornell-Larcker criterion	−0.09
CFI	0.89
TLI	0.86
RMSEA [90% CI]	0.074 [0.052–0.096]
CFit (*p* value of RMSEA ≤0.05)	0.04
SRMR	0.06
WRMR	0.88
HTMT ratio (ODI by SF-36)	
PCS [90% CI]	0.86 [0.77–0.93]
MCS [90% CI]	0.53 [0.42–0.64]
MSA	
*H*_*s*_	0.31
LCRC	0.80

Abbreviations: *ECV* explained common variance, *CR* composite reliability, *AVE* average variance extracted, *CFI* comparative fit index, *TLI* Tucker-Lewis index, *RMSEA* root mean square error of approximation, *CFit* close fit test, *SRMR* standardized root mean square residual, *WRMR* weighted root mean square residual, *HTMT* heterotrait-monotrait ratio, *SF-36* Short-Form 36 questionnaire, *PHS* Physical Health score, *MHS* Mental Health score, *PCS* Physical Component Summary, *MCS* Mental Health Component Summary, *H*_*s*_ scale scalibility, *LCRC* latent class reliability coefficient

**Table 5:** Correlation coefficients between the ODI and other scales

Scale	*r*
SF-36	
PHS	−0.64
MHS	−0.44
PCS	−0.61
MCS	−0.26
VAS	
Low back	0.38
Legs	0.36

Abbreviations: *VAS* Visual Analog Scale, *SF-36* Short-Form 36 questionnaire, *PHS* Physical Health score, *MHS* Mental Health score, *PCS* Physical Component Summary, *MCS* Mental Health Component Summary

**Table 6:** The item scalability coefficients (H_i_) of the ODI

Item	*H*_*i*_
1	0.26
2	0.24
3	0.27
4	0.34
5	0.27
6	0.36
7	0.21
8	0.32
9	0.37
10	0.41

## Discussion

Our results supported that the ODI has good reliability [7, 10]. As for the dimensionality of the ODI, our results largely opposed that the ODI is a unidimensional scale. On one hand, evidence for the ODI as a multidimensional scale was as follows: (1) ω_h_ was less than α [21]; (2) the ECV was less than 60% [22]; (3) the ODI had insufficient convergent validity; and (4) the fit statistics of the unidimensional model of the ODI were generally poor. On the other hand, our MSA shows that the ODI was a weak Mokken scale that supported the use of the raw score of the ODI as valid ordinal personal measurement of disability [7]. But a half of the ODI items performed poorly in unidimensionality (*H*_*i*_ < 0.3). Such results further signified the poor unidimensionality of the ODI [6, 8].

The ODI had good discriminant validity from the MCS and poor discriminant validity from the PCS. Such results supported that the ODI is a construct of physical disability [3]. The correlations between the ODI, the aggregate scores of the SF-36, and the VAS also supported that the ODI is a measure of physical disability.

Our results showed that the 7 items of the ODI had weak IIO property. The IIO property is a useful feature for measurement of disability. For example, if a patient with LBP reports impaired personal hygiene, the patient would also suffer from disability of the other 6 items of the ODI. Also, when a patient reports improved disability of pain intensity, the patient would have improved disability of the other 6 items of the ODI. Besides, our results concurred with recent studies that the disability of personal hygiene is the most difficult item of the ODI [7].

Our results have two implications for research of the ODI. First, the raw score of the ODI might not be the ideal aggregate score of the ODI. Alternative scoring methods of the ODI include dividing the raw score into 5 categories, and using the individual items of the ODI [3]. Second, further research should consider multidimensional scaling of the ODI, for example, multidimensional scaling and item-response theory models [41, 42].

The present study has three limitations. First, this was cross-sectional analysis of a single-site sample. We were incapable of verifying test-retest reliability of the ODI. Also, selection bias unavoidably existed. Second, the sample size was modest. Third, the original study was not designed to investigate the research questions addressed in the present study. Fourth, we did not include a reliable and valid assessment of personality disorders. Polatin et al. (1993) reported that the prevalence of at least one personality disorder was as high as 51% among chronic LBP patients [43]. Hence, as regards psychological factors of chronic LBP, it is crucial to include assessment of personality disorder. However, to the best of our knowledge, there is still a lack of a validated Chinese version of the Structured Clinical Interview for DSM-IV-TR Axis II Personality Disorders (SCID-II), which is the standard assessment of personality disorders [44]. Hence, we cannot provide the prevalence of personality disorders in the study sample.

## Conclusions

Using multiple methods, we show that the ODI was a reliable and valid scale suitable for measuring disability in patients with low back pain. But the ODI seemed to be a multidimensional scale that was against the use of the raw score of the ODI as a measurement of disability.

## Abbreviations

AVE: Average Variance Extracted
CFI: Comparative Fit Index
CFit test: Close Fit test
CR: Composite Reliability
ECV: Explained Common Variance
HTMT ratio: Heterotrait-Monotrait ratio
LBP: Low back pain
LCRC: Latent Class Reliability Coefficient
ODI: Oswestry Disability Index
RMESA: Root Mean Square Error of Approximation
SF-36: Short-Form 36 questionnaire • SF-36 subscales: - BP: Bodily Pain - GH: General Health - MH: Mental Health - PF: Physical Functioning - RE: Role-Emotional - RP: Role-Physical - SF: Social Functioning - VT: Vitality • SF-36 composite scales: - MCS: Mental Health Component Summary - MHS: Mental Health Score - PCS: Physical Component Summary - PHS: Physical Health Score
SRMR: Standardized Root Mean Square Residual
TLI: Tucker-Lewis Index
VAS: Visual Analog Scale
WRMR: Weighted Root Mean Square Residual
